# Antioxidant Supplementation with ProCloSupp Protects Against Renal Toxicity of Atypical Antipsychotics in Rats: Implications for Safer Treatment Strategies

**DOI:** 10.3390/life15111679

**Published:** 2025-10-28

**Authors:** Tanja Grahovac, Teodora Vidonja Uzelac, Zorana Oreščanin Dušić, Dušan Spasić, Milica Mijović, Aleksandra Nikolić-Kokić, Čedo Miljević, Duško Blagojević

**Affiliations:** 1 Department of Physiology, Institute for Biological Research “Siniša Stanković”—National Institute of Republic of Serbia, University of Belgrade, 11000 Belgrade, Serbia; teodora.vidonja@ibiss.bg.ac.rs (T.V.U.); zoranaor@ibiss.bg.ac.rs (Z.O.D.); dblagoje@ibiss.bg.ac.rs (D.B.); 2 Department of Pharmacology, Clinical Pharmacology and Toxicology, Faculty of Medicine, University of Belgrade, 11000 Belgrade, Serbia; dusan.spasic.dr@med.bg.ac.rs; 3 Institute of Pathology, Faculty of Medicine, University of Priština, 38220 Kosovska Mitrovica, Serbia; milica.mijovic@med.pr.ac.rs; 4 Outpatient Department, Institute of Mental Health, School of Medicine, University of Belgrade, 11000 Belgrade, Serbia; cedo.miljevic35@gmail.com

**Keywords:** clozapine, aripiprazole, risperidone, antioxidants, dietary supplement

## Abstract

Atypical antipsychotics (AAP), including clozapine (Clo), aripiprazole (Ari), and risperidone (Ris), are widely used in psychiatry but can lead to kidney damage due to oxidative stress. This study investigated whether dietary supplementation with selected antioxidants—ellagic acid, vitamin C, zinc, and seleno-methionine (SeMet) in fish oil, formulated as the composite product “ProCloSupp” (PCS)—can mitigate the oxidative damage induced by subchronic administration of AAP. Rats were treated with each antipsychotic for 28 days, with PCS added in the last 14 days. The kidney tissue was examined histologically and by determining the activities of antioxidant enzymes (copper, zinc and manganese superoxide dismutase—CuZn SOD and Mn SOD, catalase—CAT, glutathione peroxidase—GPx, glutathione reductase—GR, glutathione S-transferase—GST). All AAPs caused discrete to moderate renal damage and significant changes in enzyme profiles, which were most pronounced with Ari. Clo and Ari significantly decreased CuZn SOD and Mn SOD activity, while Ris only affected Mn SOD. Clo additionally increased CAT activity, while Ari increased GPx activity. Antioxidant-related protein levels increased only in the Ris group. PCS supplementation increased CuZn SOD and GPx activities and was associated with less pronounced histopathological changes than antipsychotic treatment alone. In conclusion, subchronic Clo, Ari, and Ris exposure induces oxidative renal damage in rats, while PCS supplementation enhances antioxidant defences and attenuates tissue damage. These results support PCS as a potential nephroprotective strategy in antipsychotic therapy.

## 1. Introduction

Atypical antipsychotics (AAPs) are a cornerstone of therapy for schizophrenia and several other psychiatric conditions, including acute mania, treatment-resistant depression, and dementia. Despite their proven efficacy [1], AAPs have been increasingly linked to systemic adverse effects. Among these, potential nephrotoxic outcomes such as acute kidney injury (AKI) and interstitial nephritis have recently attracted attention [2]. Although the mechanisms underlying AAP-induced renal injury remain unclear, growing evidence implicates oxidative stress particularly that arises from mitochondrial dysfunction as a key contributor [3]. Oxidative stress, which damages DNA, lipids, and proteins and contributes to long-term kidney dysfunction [4], was previously shown in our studies to be induced by clozapine, sertindole, and ziprasidone through altered antioxidant enzyme activity [5]. Building on these findings, we extended the analysis to two additional AAPs, aripiprazole (Ari) and risperidone (Ris), to further examine their effects on oxidative stress in renal tissue. Because oxidative processes are a major cause of tissue damage, we also explored a potential protective approach, supplementation, with ellagic acid, zinc, ascorbic acid, and seleno-methionine (SeMet) to mitigate AAP-induced oxidative stress.

Ellagic acid, known for its antioxidant and anti-inflammatory properties, reduces oxidative stress-induced kidney injury [6,7], mainly by inhibiting NF-κB activation and suppressing pro-inflammatory cytokines (TNF-α, IL-1, IL-6) in renal epithelial cells. Together with ellagic acid, ascorbic acid, SeMet, and zinc act synergistically to counteract AAP-induced oxidative stress by enhancing the activity of key antioxidant enzymes such as superoxide dismutase (SOD) and glutathione peroxidase (GPx) [8,9,10]. SeMet, an organic selenium compound, alleviates oxidative stress and inflammation, improves renal function, and protects against ammonia-induced kidney injury by increasing GPx and SOD activity [11,12,13]. Zinc, a crucial cofactor for many enzymes and transcription factors, protects cells by stabilising membranes, reducing inflammation, and stimulating metallothionein synthesis, which neutralises reactive oxygen species [14,15].

Considering these points, the main objective of this study was to investigate the effects of 28-day daily administration of atypical antipsychotics, alone or combined with 14 days of supplementation with a mixture of ellagic acid, ascorbic acid, SeMet, and zinc (ProCloSupp—PCS), on oxidative stress-induced renal dysfunction.

We hypothesised that supplementation with these agents would attenuate AAP-induced oxidative stress and thereby mitigate renal dysfunction, as assessed histopathologically and through measurements of antioxidant enzyme activity and protein levels.

## 2. Materials and Methods

### 2.1. Materials

Clo (Leponex) was provided by Mylan, Hungary, Kft), and Ari (Bipodis) was obtained from Actavis LTD, BLB016 Bulebel Industrial Estare, Zejtun, Malta) and Ris (Rispolept) (JANSSEN-CILAG S.P.A. Via C. Janssen, 04100 Borgo San Michele, Latina, Italy). Copper–zinc superoxide dismutase (CuZn SOD), manganese superoxide dismutase (Mn SOD), catalase (CAT), glutathione reductase (GR), glutathione peroxidase (GPx), and anti-beta actin levels were detected using antibodies from Abcam (ab13489, ab13533, ab16731, ab16801, ab22604, and (ab8227), respectively), secondary anti-mouse (ab97046) and anti-rabbit (ab6721) horseradish peroxidase (HRP)-linked antibodies, were also obtained from Abcam (Cambridge, UK). Composite supplement acronym “ProCloSupp” (PCS) contain Ellagic acid—hydrate, Zn cytrate, L-ascorbic acid, Se-L-Met, all provided by Sigma Aldrich, St. Louis, MO, resuspended in fish oil produced by Jekogal (Galafarm, Belgrade, Serbia). ProCloSupp is under patent protection procedure by The Intellectual Property Office of The Republic of Serbia. All other chemicals used in experimental procedures purchased from (Sigma Aldrich, St. Louis, MO, USA).

### 2.2. Animals

Sixty-four adult male Wistar albino rats (4-month-old, weighing 260–450 g) were randomly divided into eight experimental groups (*n* = 8 per group) based on body weight and litter origin to minimise biological variability. Prior to the start of the experiment, all animals underwent a 7-day acclimatization period under controlled laboratory conditions. Animals were housed in groups of 3, 3, or 2 rats per standard clear cage, with food and water available ad libitum. The procedures complied with Directive 2010/63/EU regarding the protection of animals used for experimental and other scientific purposes. The study was approved by the Ministry of Agriculture, Forestry and Water Management—Veterinary Directorate (decision number 323-07-08256/2022-05, from 25 July 2022). Rats were kept under standard laboratory conditions at 22 ± 2 °C, relative humidity of 50 ± 10%, and a 12 h light/dark cycle. Mortality and animal weights were recorded during the course of treatment as an index for antapical antipsychotic toxicity (Figure S1).

### 2.3. Drug Treatment

All drugs were prepared (water suspension of powdered tablets) and administered daily by gavage in the morning to ensure that no drug was lost. The rats were dosed according to the drug calculation formula [16]. Rats were treated daily by gavage with water (control), Clo (45 mg/kg/d), Ari (1.29 mg/kg/d), or Ris (0.53 mg/kg/d) for 4 weeks. After two weeks of treatment with the antipsychotic, we administer a daily dietary supplement (“ProCloSupp”) via a gastric tube, one hour after administration of the antipsychotic. ProCloSupp contains ellagic acid—hydrate 50 mg/kg body weight, Zn-cytrate 2 mg/kg body weight, L-ascorbic acid 10 mg/kg body weight, Se-L-Met 10 µg/kg body weight, resuspended in fish oil (1 mL cod liver oil contains vitamin A 276 µg and vitamin D 2.3 µg).

### 2.4. Tissue Collection

The animals were sacrificed by decapitation on the 28th day after an overnight fast. To ensure optimal preservation of both enzymatic activity and tissue morphology, one kidney from each animal was used for biochemical analysis and the other for histopathological analysis. The right kidneys were removed immediately, frozen in liquid nitrogen, and stored at −80 °C until further analysis. The left kidney was removed, fixed in 4% paraformaldehyde solution for 24 h, dehydrated with increasing concentrations of ethanol and xylene, and used for histopathological examination of both the renal cortex and medulla. After embedding in Histowax (Histolaboduct AB, Gothenburg, Sweden), each tissue block was sectioned to 5 µm thickness using a rotary microtome (RM2125 RT Leica Microsystems, Wetzlar, Germany).

### 2.5. Tissue Preparation and Determination of Antioxidant Enzyme Activities

To prepare whole tissue extracts, the right kidney was homogenised in 10 volumes (*w/v*) of 50 mM Tris-HCl, 0.25 M sucrose, 1 mM ethylenediaminetetraaceticacid (EDTA), pH 7.4, and sonicated for 3 × 10 s at 10 MHz (Sonopulse, Bandelin, Berlin, Germany) on ice, followed by centrifugation at 4 °C for 90 min at 105,000× *g* (Beckman L7-55 ultracentrifuge). The supernatants were used as whole tissue extracts. Total SOD activity was determined according to the adrenaline method [17]. One SOD unit was defined as the amount of enzyme required to reduce the autooxidation rate of adrenaline by 50% at a pH of 10.2. To determine Mn SOD activity, the assay was performed after preincubation with 8 mM potassium cyanide. CuZn SOD activity was calculated as the difference between total SOD and Mn SOD activity. CAT activity was determined according to Beutler [18]. One unit of CAT activity was defined as the amount of enzyme that decomposes 1 mmol H_2_O_2_ per minute at 25 °C and pH 7. GPx activity was determined by glutathione reduction in t-butyl hydroperoxide using a modification of the assay described by Paglia and Valentine [19]. One unit of GPx activity was defined as the amount of enzyme required to oxidise 1 μmol of NADPH per minute at 25 °C and pH 7. GR activity was determined according to the method of Glatzle and colleagues [20]. One unit of GR activity was defined as the amount of enzyme required to oxidise 1 μmol of NADPH per minute at 25 °C and pH 7.4. To measure the total activity of glutathione-S-transferases (GSTs), 1-chloro-2,4-dinitrobenzene (CNDB) was used as a substrate [21]. One unit of GST activity is defined as the amount of enzyme required to conjugate 1 μmol of CNDB with glutathione (GSH) per minute at 25 °C. All enzyme activities are expressed in units (U) per milligram of protein. Protein concentration was determined according to the method of Lowry and coworkers [22] using bovine serum albumin as a standard.

### 2.6. SDS Polyacrylamide Gel Electrophoresis and Immunoblotting

Proteins from whole tissue extracts were dissolved in 12% sodium dodecyl sulphate (SDS) polyacrylamide gels and then transferred to a polyvinylidene difluoride (PVDF) membrane. Unbound areas on the membranes were blocked for 1.5 h with 1% non-fat dry milk. After blocking, the membranes were incubated with a primary antibody and then with an HRP-conjugated secondary antibody. Protein load was corrected for all samples by analysing the membranes for β-actin. Immunopositive bands were visualised using the enhanced chemiluminiscent (ECL) method and quantified using the iBright FL1500 Imaging System software (version 4.0.1) (Thermo Fisher Scientific, Waltham, MA, USA).

### 2.7. Light Microscopy

After the experimental animals had been sacrificed, the left kidneys were removed for pathological analysis and macroscopically examined. Tissue samples taken from different parts of the kidney were fixed in 10% buffered formalin, embedded in paraffin blocks, sectioned with a microtome at 5 μm thickness and stained with the haematoxylin–eosin (HE) method. The histomorphological appearance of the renal cortex (epithelial cells of the proximal renal tubules, shape of the tubule lumina, and morphology of the glomeruli) and the renal medulla was interpreted by qualitative HE analysis. The toxic effects manifest themselves in a so-called parenchymal degeneration, which is accompanied by an excessive accumulation of water in the cytoplasm of the cells. This leads to a swelling of the epithelial cells, resulting in a folding of their apical parts, which are directed towards the lumen of the tubules (this explains the change in the shape of the tubule lumen, which changes from round to oval in unaffected kidneys and to star-shaped in obvious changes). The shape of the tubule lumen is therefore a direct consequence of the size, especially the water content in the epithelial cells lining the tubule lumen. The shape of the lumen is divided into two categories: 1. round or oval (when the cells have a normal volume) and 2. star-shaped (when the cell volume increases due to water accumulation). In addition, the cytoplasm of epithelial cells becomes lighter with increasing water content and the nuclei are either less coloured or absent.

A semi-quantitative method was used to characterise the shape and visibility of the epithelial cell nuclei, which led to a classification into the following categories: 1. normal and clearly visible round cell nuclei or 2. poorly visible or focally absent cell nuclei.

Based on these observations, the interpretation was summarised into two types of changes:

1. Discrete—when the shape of the tubule lumen was regular (oval or round) in at least 75% of all tubules examined and when round nuclei were present in all cells or poorly visible in less than 50% of all tubules examined.
2. Moderate—if the shape of the tubule lumen was stellate in at least 75% of all tubules examined and the nuclei were poorly visible in at least 50% of all cells examined, with focal absence in less than 50% of all tubules.

### 2.8. Statistical Analysis

The normality of data distribution was verified using the Shapiro–Wilk test prior to applying parametric analyses. A two-way ANOVA was performed for each individual antipsychotic treatment using drug (yes/no) and supplementation (yes/no) as factors, followed by Tukey’s HSD post hoc test. Data expressed as ratios or percentages were logarithmically transformed before ANOVA analysis to ensure homogeneity of variance. A significance level of *p* < 0.05 was considered statistically significant. Statistical analyses were performed using Python 3.11 (SciPy and statsmodels libraries), and data visualisation was carried out in GraphPad Prism 9.0 (GraphPad Software, San Diego, CA, USA).

## 3. Results

### 3.1. Effects of APP with or Without PCS on Kidney Morphology

**Figure 1:**
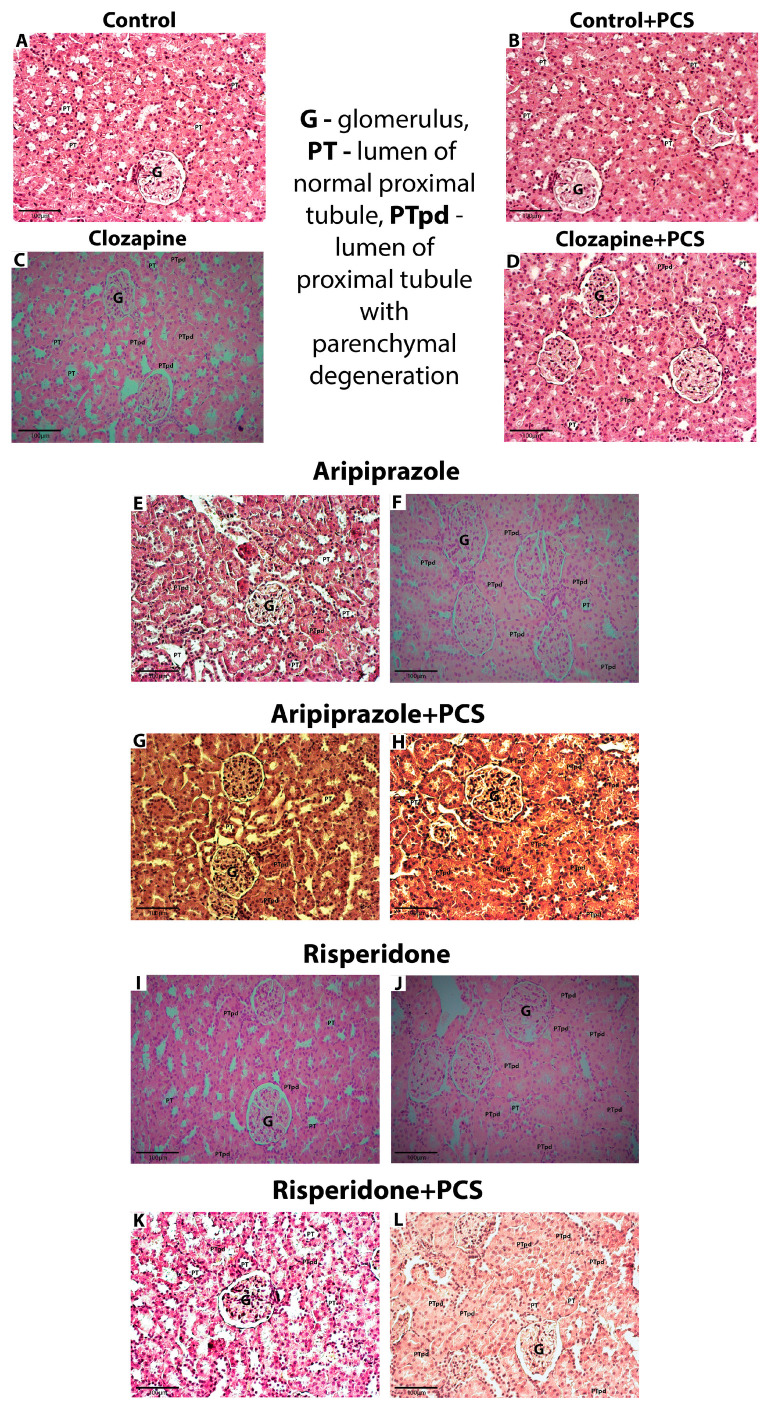
Representative micrographs showing kidney morphology in control group and groups treated with Clo, Ari, and Ris without and with supplement (H&E, 200×). (**A**,**B**)—Kidney cortex with preserved glomeruli, normal epithelial cells, and round or oval lumen of proximal tubules; (**C–E**,**G**,**I**,**K**)—discrete changes (the lumen of proximal tubules are round or oval in at least 75% of all tubules, while the volume of the tubular cells is slightly increased; nuclei are focally less visible in less than 50% of all cells); (**F**,**H**,**J**,**L**)—moderate changes (the lumen of proximal tubules are irregular, stellate in at least 75% of all tubules, while the volume of the tubular cells is markedly increased; nuclei are less visible or focally absent in at least 50% of all cells). Abbreviations in figures: G—glomerulus; PT—lumen of normal proximal tubule; PTpd—lumen of normal proximal tubule with parenchymal degeneration.

Histopathological analysis revealed normal morphology of the renal cortex and medulla in all eight animals of the control group without and with PCS supplement. The proximal tubules had a round to oval shape and the lumens showed similar characteristics. The nuclei of the epithelial cells lining these tubules were well demarcated, with the rounded ends clearly visible (Figure 1).

In contrast to controls, discrete changes [shape of the tubule lumen was regular (oval or round) in at least 75% of all tubules examined and when round nuclei were present in all cells or poorly visible in less than 50% of all tubules examined] were observed in all eight animals treated with Clo (Table 1). Histopathological analysis of the kidneys of animals treated with Clo and the supplement PCS showed the same characteristics as those of the group treated with Clo alone; discrete changes were also observed in all animals.

In the Ari group, discrete changes were observed in one animal, while moderate changes [shape of the tubule lumen was stellate in at least 75% of all tubules examined and the nuclei were poorly visible in at least 50% of all cells examined, with focal absence in less than 50% of all tubules] occurred in seven animals. The histopathological changes in the Ari + PCS group were less pronounced compared to Ari alone, with discrete changes observed in seven animals and moderate changes in only one animal.

Histopathological changes were also observed in the Ris-treated group, where 50% of the animals showed moderate changes, while the other 50% showed discrete changes. In the group treated with Ris and PCS, histopathological changes were less pronounced compared to Ris alone, with discrete changes observed in seven animals (88%) and moderate changes in one animal (12.5%).

### 3.2. Effects of AAP Treatment Without and with Supplementation with PCS on Renal Antioxidant Enzymes Function

**Figure 2:**
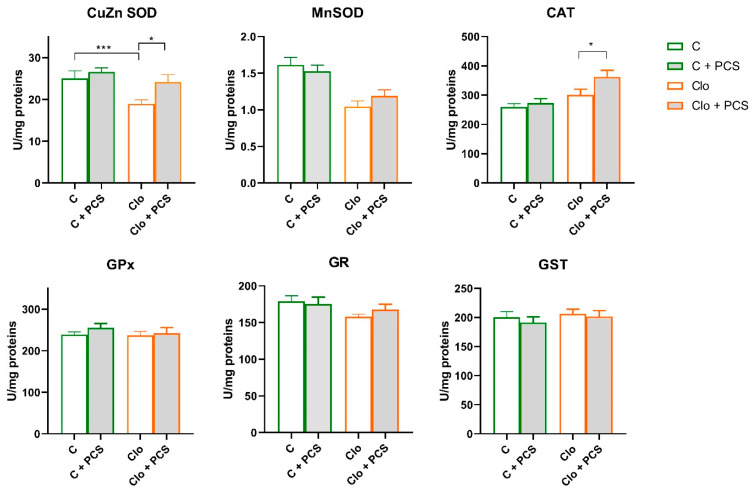
Effects of a two-week treatment with PCS on the activity of antioxidant enzymes and GST in the kidney of four-week Clo-treated rats. Enzyme activities were determined spectrophotometrically. Data are presented as means ± SEM (*n* = 8 per group) in units per milligrams of total protein. C: control group; C + PCS: control + supplement PCS; Clo: clozapine group; Clo + PCS: clozapine group + supplement PCS. Statistical significance was tested by two-way ANOVA (results described in the text) and compared post hoc by Tukey’s HSD test (results shown in the figures * *p* < 0.05 and *** *p* < 0.001).

After spectrophotometric analysis of the antioxidant enzyme activity, two-way analysis of variance (factors Clo and PCS supplementation) showed statistically significant lower both CuZn SOD (Clo effect, F = 8.37, *p* < 0.01) and Mn SOD (Clo effect, F = 26.03, *p* < 0.001) antioxidant enzyme activity in the kidneys of rats treated with Clo, accompanied by a significant increase in CAT activity (Clo effect, F = 13.43, *p* < 0.001). On the other hand, supplementation led to generally slightly but statistically significant elevated CuZn SOD activity (PCS supplementation effect, F = 5.48, *p* < 0.05) and CAT (PCS supplementation effect, F = 4.41, *p* < 0.05). Post hoc Tukey HSD test revealed that PCS supplementation, in fact, elevated CuZn SOD activity in Clo-treated rats to the level of controls and additionally increased CAT activity. There were no changes in the activities of GPx, GR, and GST (Figure 2).

**Figure 3:**
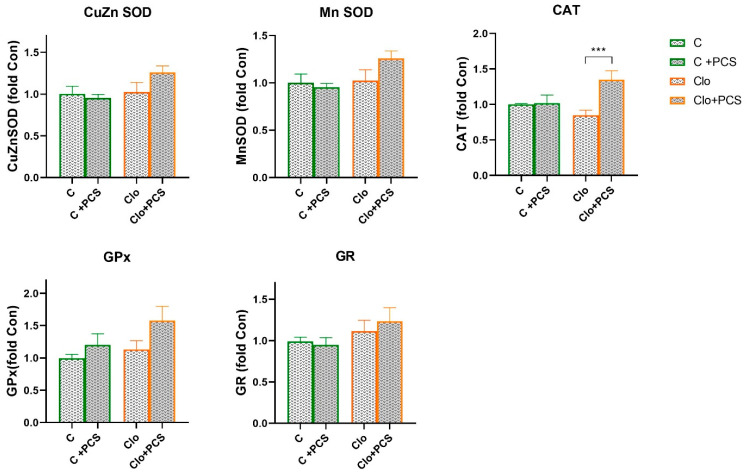
Effects of a two-week treatment with PCS on protein expression of antioxidant enzymes in the kidney of four-week control or Clo-treated rats. Whole cell extracts (50 µg protein) were subjected to SDS-PAGE and Western blotting. β-actin was used as loading control. Representative Western blots and relative quantification of antioxidant enzyme levels of control group and groups treated with Clo and PCS are in Figure S2. Values are calculated as control to sample ratio and expressed as means ± SEM (*n* = 7). Statistical significance was tested by two-way ANOVA (results described in the text) and compared post hoc by Tukey’s HSD test (results shown in the figures *** *p* < 0.001).

Statistical analyses of protein levels of CuZn SOD, Mn SOD, CAT, GPx, and GR determined by Western blot analysis showed that supplementation with PCS elevated CAT protein expression in Clo animals that received Clo (ANOVA, Supplement effect, F = 8.68, *p* < 0.01; interaction effect Clo x Supplement PCS, F = 9.84, *p* < 0.01; post hoc Tukey *t*-test *p* < 0.01), while the expression levels of other antioxidant enzymes were similar (Figure 3). The original images of Western blots are provided as Supplementary Material (Figure S1).

**Figure 4:**
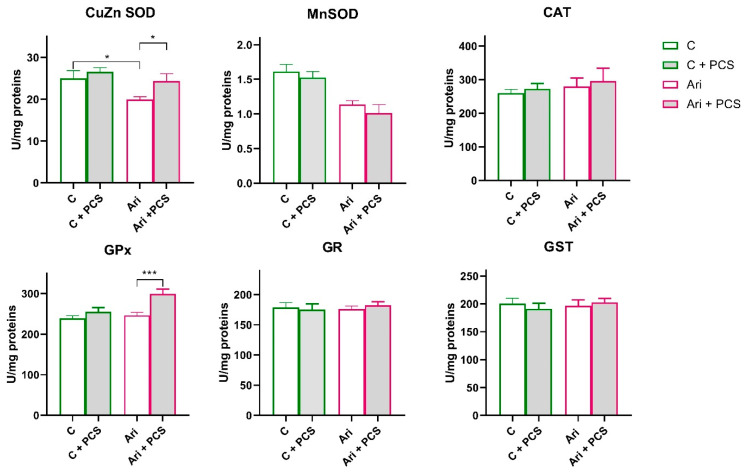
Effects of a two-week treatment with PCS on the activity of antioxidant enzymes and GST in the kidney of four-week Ari-treated rats. Enzyme activities were determined spectrophotometrically. Data are presented as means ± SEM (*n* = 8 per group) in units per milligrams of total protein. C: control group; C: control + supplement PCS; Ari: aripiprazole group; Ari + PCS: aripiprazole + supplement PCS. Statistical significance was tested by two-way ANOVA (results described in the text) and compared post hoc by Tukey’s HSD test (results shown in the figures * *p* < 0.05 and *** *p* < 0.001).

**Figure 5:**
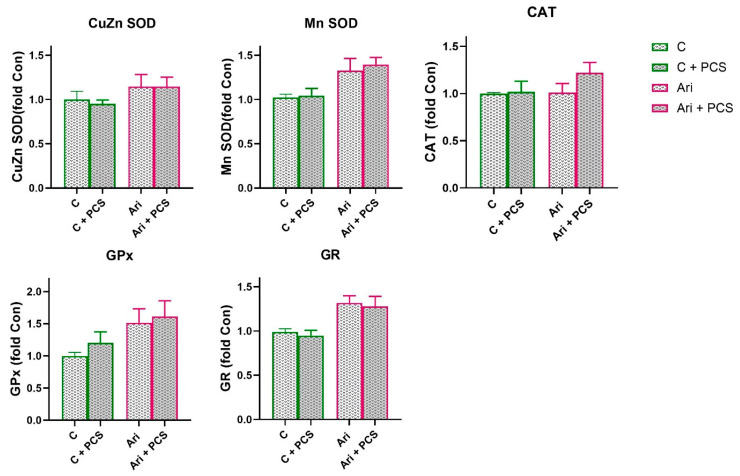
Effects of a two-week treatment with PCS on protein expression of antioxidant enzymes in the kidney of four-week control or Ari-treated rats. Whole cell extracts (50 µg protein) were subjected to SDS-PAGE and Western blotting. β-actin was used as loading control. Representative Western blots and relative quantification of antioxidant enzyme levels of control group and groups treated with Ari are in Figure S2. Values are calculated as control to sample ratio and expressed as means ± SEM (*n* = 7). Statistical significance was tested by two-way ANOVA (results described in the text) and compared post hoc by Tukey’s HSD test.

A similar trend in antioxidative enzymes activity was observed in the Ari-treated group. A significant decrease in CuZn SOD (ANOVA Ari effect, F = 6.6, *p* < 0.05) and Mn SOD (ANOVA Ari effect, F = 26.7, *p* < 0.001) activity, and elevation of GPx (ANOVA Ari effect, F = 7.6, *p* < 0.01) were observed in Ari-treated rats (Figure 3). PCS supplementation resulted in a general elevation of CuZn SOD (ANOVA Ari effect, F = 4.513, *p* < 0.05) and GPx activity (ANOVA Ari effect, F = 13.26, *p* < 0.001). Supplementation with PCS significantly restored CuZn SOD activity in the Ari-treated group and led to the increase in GPx activity (*p* < 0.001) (Figure 4). The activities of the other antioxidant enzymes were unchanged. However, Western blot analysis of protein levels of antioxidant enzymes in the kidneys of Ari-treated animals showed statistically significant increases in Mn SOD (ANOVA Ari effect, F = 14.86, *p* < 0.001), GPx (ANOVA Ari effect, F = 5.46, *p* < 0.05) and GR (ANOVA Ari effect, F = 8.21, *p* < 0.01) proteins (Figure 5). Supplementation had no statistically significant effect on antioxidative defence system. The original images of Western blots are provided as Supplementary Material (Figure S1).

**Figure 6:**
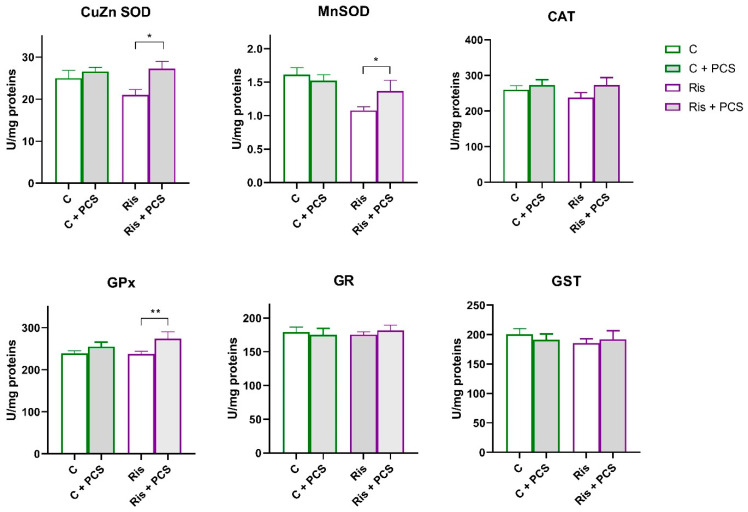
Effects of a two-week treatment with PCS on the activity of antioxidant enzymes and GST in the kidney of four-week Ris-treated rats. Effects of a two-week treatment with PCS on the activity of antioxidant enzymes and GST in the kidney of four-week control and Ris-treated rats. Enzyme activities were determined spectrophotometrically. Data are presented as means ± SEM (*n* = 8 per group) in units per milligrams of total protein. C: control group; C: control + supplement PCS; Ris: risperidone group; Ris + PCS: risperidone + supplement PCS. For transparency and clarity, the numerical values corresponding to the graphical data are shown in the Supplementary Table S1. Statistical significance was tested by two-way ANOVA (results described in the text) and compared post hoc by Tukey’s HSD test (results shown in the figures * *p* < 0.05; and ** *p* < 0.01).

**Figure 7:**
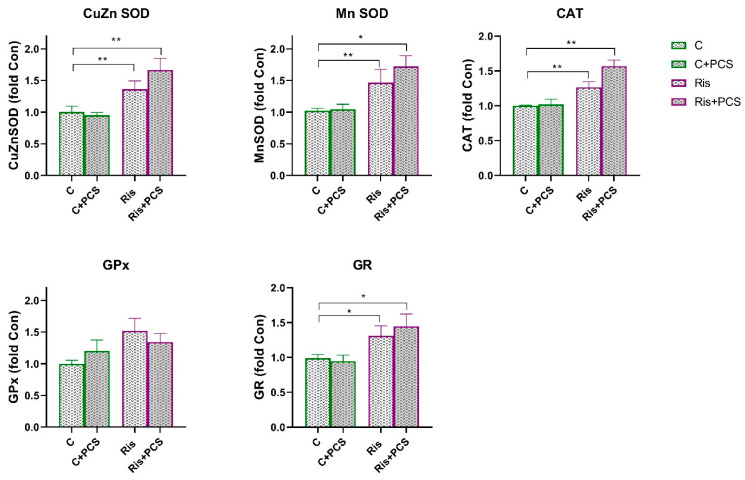
Effects of a two-week treatment with PCS on protein expression of antioxidant enzymes in the kidney of four-week control or Ris-treated rats. Whole cell extracts (50 µg protein) were subjected to SDS-PAGE and Western blotting. β-actin was used as loading control. Representative Western blots and relative quantification of antioxidant enzyme levels of control group and groups treated with Ari are in Figure S2. Values are calculated as percentage of control and expressed as means ± SEM (*n* = 7). Statistical significance was tested by two-way ANOVA (results described in the text) and compared post hoc by Tukey’s HSD test (results shown in the figures * *p* < 0.05 and ** *p* < 0.01).

In rats treated with Ris, Mn SOD activity was significantly reduced compared to control both naive and supplemented rats and (ANOVA Ris effect, F = 11.12, *p* < 0.01; Tukey’s HSD post hoc *t*-test, *p* < 0.01 and *p* < 0.05, respectively). However, supplementation with PCS led to elevation of Mn SOD activity to the level of controls (Figure 6). Analysis of variance showed that supplementation elevated the activities of Ris-treated rats, a statistically significant increase in the activity of CuZn SOD (ANOVA supplementation effect, F = 6.72, *p* < 0.05) and GPx (ANOVA supplementation effect, F = 5.62, *p* < 0.05), but this effect is pronounced in Ris-treated rats (Tukey’s HSD post hoc *t*-test, statically significant difference between Ris-treated rats without and with supplement, *p* < 0.05) (Figure 5). Western blot analysis of antioxidant enzyme protein levels in the kidneys of Ris-treated rats showed an overall increase with statistically significant increases in all antioxidant enzymes analysed: CuZn SOD (ANOVA Ris effect, F = 20.8, *p* < 0.001), Mn SOD (ANOVA Ris effect, F = 14.4, *p* < 0.001), CAT (ANOVA Ris effect, F = 18.9, *p* < 0.001), GPx (ANOVA Ris effect, F = 6.46, *p* < 0.05), and GR (ANOVA Ris effect, F = 13.1, *p* < 0.001) (Figure 7). The elevation of CuZn SOD, CAT, GR, and Mn SOD were more pronounced in animals that received both Ris and supplement than animals that in Ris non-treated animals (Tukey’s post hoc HSD test). The original images of Western blots are provided as Supplementary Material (Figure S1).

## 4. Discussion

Our study demonstrates that subchronic administration of clozapine (Clo), aripiprazole (Ari), and risperidone (Ris) in rats induces discrete to moderate morphological changes in the renal cortex and medulla. Supplementation with a unique combination of antioxidants and pro-antioxidants, formulated as the composite dietary supplement ProCloSupp, ameliorated these adverse effects associated with subchronic AAP treatment (Figure 1). However, functional kidney parameters such as serum creatinine and urea were not assessed in this study. Although cases of AAP-induced renal dysfunction have been reported, the molecular mechanisms underlying kidney impairment remain poorly understood. Our findings confirm that oxidative stress may play a role in AAP-induced nephrotoxicity and further suggest that each drug has a distinct oxidative mechanism and cellular antioxidant response. None of the AAPs examined acts as a direct oxidant; rather, their oxidative effects appear linked to distinct molecular targets, regulatory pathways, and the renal impairments reported during clinical use. Moreover, the pharmacology of AAPs is complex, as each compound binds multiple receptor classes with varying affinities. This receptor diversity implies tissue-specific effects. Clozapine is considered a D4 antagonist; however, in mouse kidneys, the absence of D3 and D4 receptors leads to hypertension that is not accompanied by increased oxidative stress [23,24].

Because different reactive oxygen species (ROS) are generated across tissue compartments and contribute collectively to oxidative stress, an effective antioxidant intervention must target multiple pathways and therefore include complementary components.

Our previous findings on AAP-induced renal changes, specifically reduced CuZnSOD and MnSOD activities [5], are confirmed here and further demonstrate that the reduction reflects enzymatic inhibition rather than decreased protein levels. This inhibition is likely caused by excessive hydrogen peroxide (H_2_O_2_), which is known to inhibit SOD activity. Consequently, SOD inhibition results in increased superoxide accumulation within affected cellular compartments. Moreover, clozapine can be oxidised by NADPH oxidase, another cellular source of superoxide, to a reactive nitrenium ion that binds irreversibly to cellular components [25,26]. Accordingly, our strategy aimed to reduce excess superoxide through direct scavengers such as ascorbic and ellagic acid, while strengthening antioxidant defences via selenium supplementation to support GPx activity and facilitate the removal of H_2_O_2_ and lipid peroxides, with CAT responding to higher H_2_O_2_ levels. In addition, there are cases of Clo-induced acute interstitial nephritis/acute renal failure [27,28] that showed a significant inflammatory response and that Clo-induced acute renal failure fulfils most of the criteria for secondary hypersensitivity immune reactions. In addition, there are data on the involvement of other antipsychotics in renal impairment (Ari, Ris in combination) [29,30]. Literature data show that the association between the use of second-generation antipsychotics and the subsequent risk of chronic kidney disease was highest for Clo, lowest for Ris, and absent for Ari [31]. Recently, it was shown that Clo activates inflammasomes and that the oxidation of Clo to a reactive metabolite by myeloperoxidase is crucial for triggering the inflammatory response to Clo [32]. Therefore, in addition to antioxidants, anti-inflammatory agents such as ellagic acid, zinc, and cod liver oil are mixed in ProCloSupp to prevent both general and local inflammatory processes. Ellagic acid is not only a direct antioxidant, but also a metal chelator and an inducer of antioxidant enzyme activity, which have a strong anti-inflammatory effect both in vitro and in vivo [33].

The most significant adverse effects of clozapine are metabolic disturbances, such as weight gain, glucose dysregulation, impaired glucose tolerance, obesity, metabolic syndrome, and diabetes mellitus, all of which increase the risk of chronic kidney disease [34]. In obese mice, treatment with Clo led to glomerulonephritis, which was associated with an infiltration of inflammatory cells, a reduced activity of antioxidant enzymes (SOD, CAT, GPx), and an increased concentration of ROS in the kidney [3].

In our study, Clo did not lead to weight gain in the treated animals (Figure S1). However, histopathological analysis revealed discrete to moderate renal damage associated with a marked reduction in CuZn SOD and Mn SOD activities and a significant increase in CAT activity. Western blot analysis confirmed increased CAT protein expression, consistent with adaptive upregulation in response to high H_2_O_2_ levels. Clo is thus closely associated with oxidative stress, primarily by altering the balance of antioxidant enzymes and increasing ROS production [4,35], which is consistent with previous findings of Clo-induced renal damage [5].

Given the oxidative stress observed in clozapine-treated rats, introducing agents that reduce oxidative burden is a rational strategy to mitigate renal injury. Dietary antioxidants neutralise ROS and protect cellular structures from oxidative damage [36]. A diet rich in polyphenols, vitamin C, vitamin E, and trace elements such as zinc, copper, and selenium are widely recognised for its protective role against oxidative stress [37,38,39]. Among these, pomegranate (*Punica granatum* L.) stands out for its high polyphenol content, including ellagitannins, ellagic acid, gallic acid, and anthocyanins, which have demonstrated nephroprotective properties. For example, Sancaktutar and coworkers [40] showed that pomegranate extract reduced histological and functional kidney damage in a model of renal ischaemia–reperfusion, while Mirhan et al. [41] demonstrated protection through inhibition of the NF-κB signalling pathway and reduction in oxidative stress, neutrophil infiltration, and pro-inflammatory cytokine release. These findings are directly relevant to our study, as they provide a mechanistic rationale for the observed potential protective effects of our antioxidant supplementation, PCS, which contains ellagic acid as a key component, against AAP-induced renal oxidative stress.

In our study, PCS supplementation containing ellagic acid, vitamin C, zinc, and SeMet in a fish oil base preserved CuZnSOD activity at control levels and further increased CAT activity in clozapine-treated rats. These results suggest that PCS enhances the intrinsic antioxidant defences of the kidneys and can attenuate oxidative stress. However, PCS supplementation did not completely prevent structural kidney damage (discrete histopathological changes were found to a similar extent regardless of supplementation), suggesting that although biochemical and antioxidant defences are strengthened, they may not be sufficient to completely reverse discrete morphological damage under oxidative stress conditions in the time frame we observed.

Taken together, the changes in CuZnSOD activity observed in clozapine-treated rats were likely due to H_2_O_2_-mediated inhibition [42] which was reversed by PCS supplementation. Significant decrease in CuZnSOD activity without changes in its protein content clearly suggests that the inhibition is not due to reduced protein synthesis but rather to post-translational inactivation of the enzyme. This inhibition is mediated by high levels of ROS, particularly H_2_O_2_, which is generated in excess during clozapine metabolism and oxidative stress. H_2_O_2_ is a well-known inhibitor of both CuZnSOD and MnSOD activities, as it can oxidatively modify their active sites, reducing enzymatic function. Thus, while protein expression remains stable, enzyme activity is compromised due to ROS-induced post-translational modification, rather than intrinsic defects in the antioxidant enzymes.

In the Clo + PCS group, both enzyme activity and protein levels increased. This could be explained by the direct potential antioxidant properties of ellagic acid and ascorbic acid in PCS, which reduce superoxide availability and thereby alleviate product inhibition. Taken together, it seems that Clo provokes significant elevation of hydrogen peroxide that cannot be eliminated by both GPx and CAT activities and resulted in CuZn SOD inhibition. Addition of PCS supported ROS removal by direct potential antioxidant action, that on the other hand relax CuZn SOD inhibition and further enable Clo + PCS animals to elevate CAT-mediated hydrogen peroxide removal.

CAT activity significantly increased in the clozapine group compared with controls, and PCS supplementation further enhanced both CAT activity and protein expression in the Clo + PCS group. This indicates that PCS can stimulate the de novo synthesis of CAT protein in addition to post-translational effects. The increased CAT activity and expression probably represent an adaptive response to increased H_2_O_2_ concentrations. Polyphenols from PCS, especially ellagic acid, are known modulators of redox-sensitive signalling pathways (e.g., Nrf2/ARE) [43], which could explain the induction of transcription of antioxidant enzymes, including CAT. Such adaptation complements the restoration of CuZn SOD activity and contributes to a more efficient clearance of reactive oxygen species, ultimately reducing oxidative stress in the kidney.

Although aripiprazole is generally considered less nephrotoxic, in our study it produced the most pronounced histopathological alterations (one discrete and seven moderate cases) among the tested antipsychotics. This was accompanied by a significant reduction in CuZn SOD and Mn SOD activities. Since this reduction in activity was not accompanied by a reduced enzyme protein content (the Mn SOD protein content is even increased), the inhibition of activity is post-translational. Again, the inhibition appears to be due to hydrogen peroxide, which is supported by the fact that supplementation with PCS significantly increased CuZn SOD activity in Ari-treated animals, reaching the level observed in control animals, which was also accompanied by an increase in GPx activity. This suggests that the improvement occurs at the level of increased removal of superoxides and peroxides and restoration of redox balance. These results are consistent with previous findings indicating Ari-induced oxidative stress in renal tissue [44]. In addition, our results showed significant protective effects of PCS supplementation on kidney morphology and less frequency of the negative effects of Ari (more discrete than moderate changes).

Although several studies highlight the beneficial effects of Ari in improving oxidative imbalance, especially in ischaemia–reperfusion models, partly due to nitric oxide modulation associated with Ari itself [45], our results showed negative effects on kidney morphology. If we assume its protective effect on kidney ischaemia–reperfusion, it could be that Ari leads to impaired NO-driven processes and, consequently, to the production of reactive nitrogen species with chronic treatment. However, its long-term effects on the kidneys and oxidative stress conditions need further investigation, but a combined antioxidant supplementation, in our case with PCS, proved to be very beneficial.

In the aripiprazole group, CuZnSOD inhibition without corresponding changes in protein expression again suggests elevated hydrogen peroxide levels and consequent enzymatic inhibition. However, the elevation of GPx activity (again without statistically significant changes in protein expression) in Ari + PCS suggests lesser state of hydrogen peroxide production in Ari animals compared to Clo, and GPx as defending mechanisms (GPx enzyme operates in lower peroxide concentration compared to CAT) [46]. Elevation of GPx activity can be due to supplementation with Se as a part of PCS, as well as involvement of lipid peroxides in oxidant action of Ari.

In the case of GPx, the activity remained unchanged in the Ari group, but increased significantly in Ari + PCS in contrast to the Clo group. This reflects the different oxidative profile of Ari, which induces more lipid peroxides and less H_2_O_2_. GPx, which is effective at low peroxide concentrations and in detoxifying lipid peroxides, is therefore more relevant here. The increase with PCS is probably due to the fact that selenium supplementation (SeMet) increases GPx activity and the protective effect of PCS polyphenols prevents oxidative inactivation. In CuZn SOD, the activity decreased under Ari treatment despite stable protein levels, indicating post-translational inhibition by peroxides. With Ari + PCS, the activity was restored without changes in protein levels, indicating an abolition of product inhibition by PCS antioxidants.

Overall, these findings indicate that aripiprazole induces a distinct oxidative profile compared with clozapine, characterised by greater lipid peroxidation and lower H_2_O_2_ accumulation. PCS counteracts this by increasing GPx activity through selenium support and antioxidant protection and restoring CuZn SOD function through ROS filtering.

Cases of risperidone-associated renal damage have been reported [47,48], although the underlying mechanisms remain poorly understood. Histopathological examination in our study in rats revealed moderate and discrete changes after subchronic 4-week treatment with Ris. PCS supplementation showed a shift in the frequency of discrete than moderate renal histopathology in this group and resulted in a significant increase in the protection of antioxidant enzyme activity, particularly restoring CuZn SOD and Mn SOD activities to the level of the control group, in addition to GPx activity, which improved antioxidant protection. However, Western blot analyses showed that Ris induced an increase in the protein content of all antioxidant enzymes investigated. This induction did not lead to an increase in their activities, indicating strong inhibition. Furthermore, the increase induced by Ris is more pronounced in animals that had received a dietary supplement. This suggests that some of the similar cellular antioxidant defence mechanisms acting against Ris-mediated redox changes are supported by supplementation with ProCloSupp (superoxide and H_2_O_2_ scavengers, selenium support for GPx). In each case, supplementation had a significant effect on improving the renal toxicity of Ris by helping renal cells to restore redox balance. These results are consistent with previous findings on Ris-induced nephrotoxicity. Bilgiç and colleagues [49] demonstrated that resveratrol, known for its antioxidant properties, provides protection against Ris-induced renal damage through oral supplementation. In line with these findings, the current results show that PCS supplementation has a protective effect in Ris-treated rats, so it is not surprising that supplementation of Ris-treated rats with PCS achieves such good results.

Thus, protein levels of CuZn SOD, Mn SOD, and CAT increased with Ris alone, but this was not accompanied by higher activity, again indicating oxidative inhibition. With Ris + PCS, protein expression increased further, likely due to antioxidant signalling (e.g., Nrf2 activation), but activity did not fully recover within the experimental period. These results suggest that Ris induces oxidative stress primarily through increased protein synthesis without corresponding functional activation, while PCS promotes expression and partial protection but cannot fully reverse oxidative impairment.

Our results indicate that each AAP triggers oxidative stress through distinct, drug-specific pathways. Selected AAPs differ in their mechanisms of action, pharmacokinetics, and ability to disturb redox homeostasis. Therefore, different types of ROS are likely affected differently depending on the AAP. Clo appears to predominantly increase hydrogen peroxide-related oxidative stress, while Ari seems to have a greater impact on pathways associated with lipid peroxidation, as inferred from changes in the activity of antioxidant enzymes, although ROS were not directly measured. Ris, on the other hand, appears to broadly modulate ROS-related pathways affecting all analysed enzymes. Our finding that the activity of GST (the most important detoxification enzyme after Cyt450 activity) was not altered suggests that subchronic treatment with AAP has no effect on the chemical environment of the kidneys. A limitation of this study is the relatively short PCS supplementation period: two weeks of pretreatment with AAPs followed by two weeks of co-treatment, aimed primarily at assessing early oxidative and histopathological effects. However, extending the duration of PCS administration might provide further information on its long-term protective or reparative effects on renal tissue. Despite this limitation, PCS, a unique combination of antioxidants and pro-antioxidants, significantly attenuated AAP-induced histopathological changes and provided protection even when antipsychotic therapy was already established. Through its multi-component formulation, ProCloSupp targets multiple oxidative mechanisms from attenuating inflammation (via ellagic acid, cod liver oil, and zinc) to stabilising antioxidant defences through GPx support (selenium) and direct radical scavenging (ascorbic acid), thereby offering a potentially safer and more effective adjunct to AAP therapy.

## 5. Conclusions

Our findings demonstrate that clozapine, aripiprazole, and risperidone exhibit nephrotoxic potential, as indicated by altered renal antioxidant enzyme activities and histopathological changes. PCS supplementation exerted a potential protective antioxidant effect, mitigating oxidative stress-related damage to varying degrees depending on the specific antipsychotic. Overall, PCS improved renal antioxidant defence and attenuated histopathological alterations, supporting its potential as an adjunct strategy to reduce AAP-induced renal oxidative injury.

## Figures and Tables

**Table 1:** Histopathological changes in AAP-treated animals without or with PCS. The percentage of moderate histopathological changes was shown.

	AAP Treated	%	AAP + ProCloSupp	%
Clo	Discrete (8/8)	100D	Discrete (8/8)	100D
Aripiprazole	Discrete (D) (1/8); moderate (M) (7/8)	12.5D; 87.5M	Discrete (7/8); moderate (1/8)	87.5D; 12.5M
Risperidone	Discrete (4/4); moderate (4/4)	50D; 50M	Discrete (7/8); moderate (1/8)	87.5D; 12.5M

## Data Availability

Data are available at https://radar.ibiss.bg.ac.rs/handle/123456789/7672.

## Supplementary Materials

The following supporting information can be downloaded at: https://www.mdpi.com/article/10.3390/life15111679/s1, Figure S1. Body weight of rats at the beginning (initial) and at the end (final) of the 28-day treatment period across all experimental groups. Abbreviations: C, control group; C + PCS, control + PCS supplementation; Clo, clozapine group; Clo + PCS, clozapine + PCS supplementation; Ari, aripiprazole group; Ari + PCS, aripiprazole + PCS supplementation; Ris, risperidone group; Ris + PCS, risperidone + PCS supplementation. Data are presented as mean ± standard error (SE). Table S1. Effects of two-week PCS supplementation on the activity of antioxidant enzymes and GST in the kidneys of rats treated for four weeks with clozapine, aripiprazole, or risperidone. Enzyme activities were determined spectrophotometrically. Data are expressed as mean ± SEM (*n* = 8 per group) in units per milligram of total protein. Abbreviations: C, control group; C + PCS, control + PCS supplementation; Clo, clozapine group; Clo + PCS, clozapine + PCS supplementation; Ari, aripiprazole group; Ari + PCS, aripiprazole + PCS supplementation; Ris, risperidone group; Ris + PCS, risperidone + PCS supplementation. Statistical significance was assessed by two-way ANOVA, with results described in the text. Figure S2. The original images of Western blots, SOD: superoxide dismutase, CAT: catalase, GPx: glutathione peroxidase, GR: glutathione reductase, 1: control group, 2: Clo group, 3: aripiprazole group, 4: risperidone group, 5: control + supplement group, 6: Clo + supplement group, 7: aripiprazole + supplement group, 8: risperidone + supplement group.

## Abbreviations

The following abbreviations are used in this manuscript:

AAPs	Atypical antipsychotics
PCS	Composite dietary antioxidant supplement “ProCloSupp”
Clozapine	Clo
Ari	Aripiprazole
Ris	Risperidone
SeMet	Seleno-methionine
GPx	Glutathione peroxidase
SOD	Superoxide dismutase
CAT	Catalase
GR	Glutathione reductase
GST	Glutathione S-transferase
ROS	Reactive oxygen species
U	Units
SDS	Sodium dodecyl sulphate
PVDF	Polyvinylidene difluoride
ECL	Enhanced chemiluminiscent
HE	Haematoxylin–eosin
