# Supplementary material for: Antioxidant Supplementation with ProCloSupp Protects Against Renal Toxicity of Atypical Antipsychotics in Rats: Implications for Safer Treatment Strategies
Source: Life (Basel). 2025 Oct 28;15(11):1679. doi: 10.3390/life15111679 (PMC12653033; doi:10.3390/life15111679)
Supplement: Supplementary file 1 [file life-15-01679-s001.zip › Figure S1.pdf]

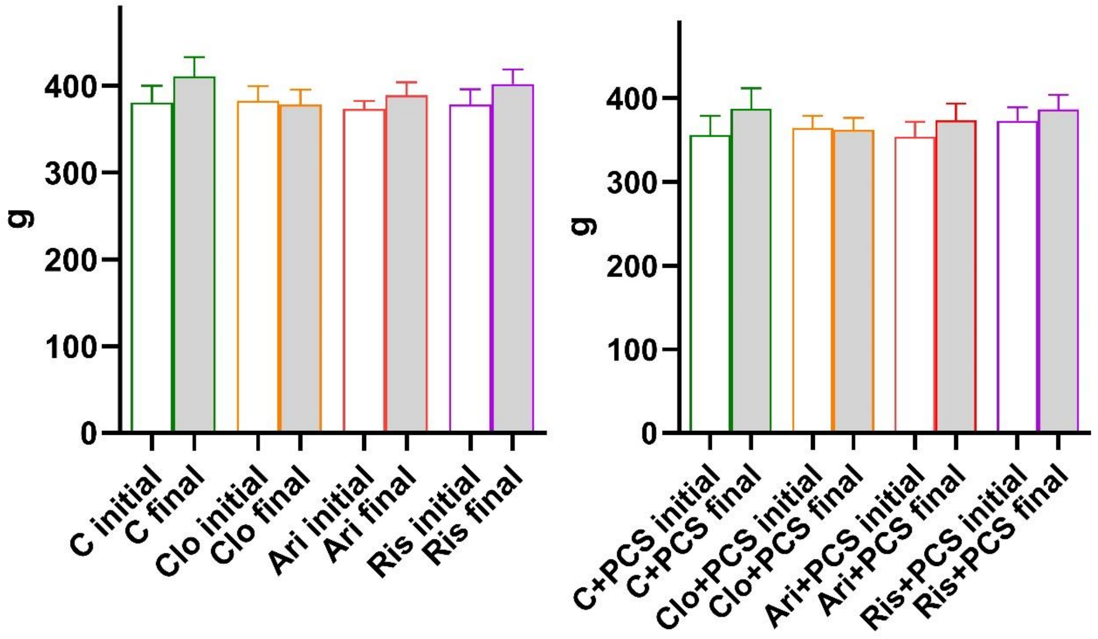


Figure S1. Body weight of rats at the beginning (initial) and at the end (final) of the 28-day treatment period across all experimental groups. Abbreviations: C, control group; C + PCS, control + PCS supplementation; Clo, clozapine group; Clo + PCS, clozapine + PCS supplementation; Ari, aripiprazole group; Ari + PCS, aripiprazole + PCS supplementation; Ris, risperidone group; Ris + PCS, risperidone + PCS supplementation. Data are presented as mean ± standard error (SE).
