# Supplementary material for: Antioxidant Supplementation with ProCloSupp Protects Against Renal Toxicity of Atypical Antipsychotics in Rats: Implications for Safer Treatment Strategies
Source: Life (Basel). 2025 Oct 28;15(11):1679. doi: 10.3390/life15111679 (PMC12653033; doi:10.3390/life15111679)
Supplement: Supplementary file 1 [file life-15-01679-s001.zip › Figure S2.pdf]

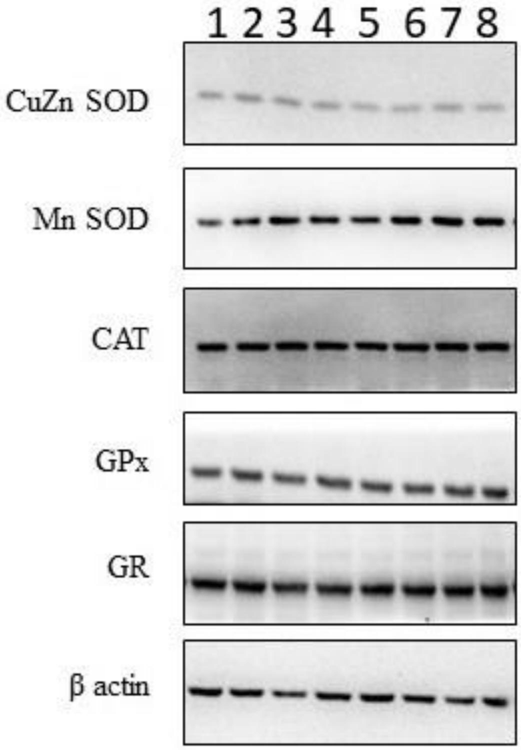

Figure S2. The original images of western blots, SOD: superoxide dismutase, CAT: catalase, GPx: glutathione peroxidase, GR: glutathione reductase, 1: control group, 2: clozapine group, 3: aripiprazole group, 4: risperidone group, 5: control + supplement group, 6: clozapine + supplement group, 7: aripiprazole + supplement group, 8: risperidone + supplement group
