# Supplementary material for: Antioxidant Supplementation with ProCloSupp Protects Against Renal Toxicity of Atypical Antipsychotics in Rats: Implications for Safer Treatment Strategies
Source: Life (Basel). 2025 Oct 28;15(11):1679. doi: 10.3390/life15111679 (PMC12653033; doi:10.3390/life15111679)
Supplement: Supplementary file 1 [file life-15-01679-s001.zip › Table S1.pdf]

Table S1. Effects of two-week PCS supplementation on the activity of antioxidant enzymes and GST in the kidneys of rats treated for four weeks with clozapine, aripiprazole, or risperidone. Enzyme activities were determined spectrophotometrically. Data are expressed as mean ± SEM (n = 8 per group) in units per milligram of total protein. Abbreviations: C, control group; C + PCS, control + PCS supplementation; Clo, clozapine group; Clo + PCS, clozapine + PCS supplementation; Ari, aripiprazole group; Ari + PCS, aripiprazole + PCS supplementation; Ris, risperidone group; Ris + PCS, risperidone + PCS supplementation. Statistical significance was assessed by two-way ANOVA, with results described in the text.

	**Control**	**Control+PCS**		**Clo**	**Clo+PCS**		**Ari**	**Ari + PCS**		**Ris**	**Ris + PCS**
**CuZnSOD**	24.97 ± 1.87	26.55 ± 1.00	Clo	18.91 ± 0.99	24.16 ± 1.73	Ari	19.92 ± 0.68	24.34 ± 1.73	Ris	21.01±1.27	27.25 ± 1.71
**MnSOD**	1.61 ± 0.10	1.52 ± 0.08		1.04 ± 0.07	1.18 ± 0.08		1.13 ± 0.05	1.00 ± 0.11		1.07 ± 0.05	1.36 ± 0.14
**CAT**	259.57 ± 11.44	272.56 ± 15.75		300.45 ± 19.71	362.23 ± 22.38		279.75 ± 25.01	295.59 ± 38.50		224.56 ± 14.12	264.86 ± 20.73
**GPx**	178.96 ± 7.64	254.73 ± 10.12		157.79 ± 3.68	167.57 ± 7.54		246.41 ± 6.62	298.95 ± 11.28		237.26 ± 6.62	273.39 ± 16.74
**GR**	238.82 ± 6.37	175.21 ± 9.54		237.04 ± 9.64	241.59 ± 14.17		176.33 ± 4.67	182.53 ± 5.83		174.24 ± 4.17	177.13 ± 7.81
**GST**	200.23 ± 9.21	191.04 ± 9.37		206.12 ± 8.22	201.50 ± 10.31		196.34 ± 10.23	202.60 ± 6.51		185.37 ± 7.56	191.71 ± 14.77
